# Risk Assessment for the Establishment of *Vespa mandarinia* (Hymenoptera: Vespidae) in the Pacific Northwest, United States

**DOI:** 10.1093/jisesa/ieab052

**Published:** 2021-08-09

**Authors:** Erik D Norderud, Scott L Powell, Robert K D Peterson

**Affiliations:** Department of Land Resources and Environmental Sciences, Montana State University, 334 Leon Johnson Hall, Bozeman, MT 59717-3120, USA

**Keywords:** honey bee, *Apis mellifera*, Asian giant hornet, risk analysis, invasive species

## Abstract

The recent introduction of the Asian giant hornet, *Vespa mandarinia* Smith, in the United States in late 2019 has raised concerns about its establishment in the Pacific Northwest and its potential deleterious effects on honey bees, *Apis* spp., and their pollination services in the region. Therefore, we conducted a risk assessment of the establishment of *V. mandarinia* in Washington, Oregon, Montana, and Idaho on a county-by-county basis. Our highly conservative tier-1 qualitative and semiquantitative risk assessment relied on the biological requirements and ecological relationships of *V. mandarinia* in the environments of the Pacific Northwest. Our risk characterization was based on climate and habitat suitability estimates for *V. mandarinia* queens to overwinter and colonize nests, density and distribution of apiaries, and locations of major human-mediated introduction pathways that may increase establishment of the hornet in the counties. Our results suggest that 32 counties in the region could be at low risk, 120 at medium risk, and 23 at high risk of establishment. Many of the western counties in the region were estimated to be at the highest risk of establishment mainly because of their suitable climate for queens to overwinter, dense forest biomass for nest colonization, and proximity to major port and freight hubs in the region. By design, our tier-1 risk assessment most likely overestimates the risk of establishment, but considering its negative effects, these counties should be prioritized in ongoing monitoring and eradication efforts of *V. mandarinia*.

Biological invasions can be ecologically and economically damaging phenomena that occur in environments around the world. The introduction and establishment of non-native species may disrupt native flora and fauna and their ecosystems and concomitantly may cause deleterious consequences to a host of economic sectors and at times even public health and safety. For example, emerald ash borer, *Agrilus planipennis* Fairmaire, a buprestid native to Asia was accidentally introduced into North America in 2002 and has since killed millions of ash trees and incurred billions of dollars of economic damage across a number of sectors (Herms and McCullough 2014).

The recent accidental introduction of the Asian giant hornet, *Vespa mandarinia* Smith, is one example of an invasive species that poses risks to economic and ecological sectors in the United States. *V. mandarinia* is the largest hornet species in the world and is a primary predator of honey bees, *Apis* spp. (Smith-Pardo et al. 2020). It was first detected in North America in Vancouver, British Columbia and then later in the United States in Whatcom County in northern Washington State in late 2019. It was detected again in May 2020 in the same county, indicating the possibility of wider spread establishment rather than just a chance introduction and detection. Further confirmed sightings since May 2020 have prompted federal and state agricultural officials to initiate eradication programs for the pest because of the insect’s propensity to decimate honey bee populations and affect human safety with its stings.

Risk assessments are often used to frame the potential impacts that invasive species pose to ecosystems, economic sectors and industry, and human health and safety. To characterize these consequences, risk assessments are regularly developed to frame the problem and ultimately to confer the degree of risk to regulatory agencies and industry pertaining to the establishment of the particular invading species. The type of risk assessment used is ultimately dependent on the data available for a particular stressor. In cases of a new introduction of an invasive species, where an invasion is in its early onset, tier-1 qualitative and semiquantitative based risk assessments are often employed as a direct result of a lack of quantitative and distribution data for the biological invader in question (Soliman et al. 2010, 2014). Tier-1 risk assessments are characterized by deliberate assumptions that represent overestimates of effect and exposure so that the resulting assessment will be conservative and err on the side of safety (SETAC 1994).

With these risks in mind, it is critical to properly frame them to be able to effectively mitigate these hazards to avoid deleterious effects to the environment and economy. Therefore, we performed a risk assessment of the establishment of *V. mandarinia* in the Pacific Northwest, focusing on Washington, Oregon, Montana, and Idaho.

## Risk Assessment

Risk assessment is a formalized process for the objective evaluation of risk in which assumptions and uncertainties are considered and presented (NRC 1983). Although precise steps and terminology vary, risk assessments typically follow the following steps: (1) problem formulation, (2) analysis phase, and (3) risk characterization. The analysis phase consists of effect and exposure assessments (NRC 1983, SETAC 1994, NRC 1996). Here, using terminology from EPA (1998), we provide a problem formulation (which establishes the goals, breadth, and focus of our assessment), an analysis phase (which has an effects assessment and an exposure assessment), and a risk characterization (which is a consideration of the joint property of effect and exposure to determine risk or what additional data are needed to calculate risk or refine risk estimates).

### Problem Formulation

The first step of any risk assessment should begin with the initial problem formulation. The problem formulation sets the stage in terms of the scope, steps, and methods of the risk assessment, delineating the ‘stressor’ and its ‘effects’ at its center of focus. In the case of our risk assessment, that stressor is *V. mandarinia* in the U.S. Pacific Northwest and its deleterious effects on the region’s ecosystems and economy. Accordingly, our establishment risk assessment begins with the known biological and ecological characteristics of the stressor, *V. mandarinia*. Additionally, the stressor description also classifies the effect that *V. mandarinia* has on its surrounding ecosystems, focusing on risks to honey bee populations and apiaries. We analyzed the extent of these effects to assess the degree of exposure to these risks in the effects and exposure assessment section of the risk assessment, which primarily analyzed climate and habitat suitability for the insect, factors influencing introduction, and risk to honey bee populations. The final section of our risk assessment drew from the findings of the previous steps, and we ultimately characterized the risks of the establishment of the *V. mandarinia* in the U.S. Pacific Northwest using a risk-rating system.

### Stressor Description

The Asian giant hornet is prevalent throughout Asia, with its range extending from mainland Asia into Taiwan, Japan, and South Korea (Archer 1995). The insect is in the Vespidae family, within the order Hymenoptera. *V. mandarinia* is the largest known species of hornet in the world, ranging from 38 to 50 mm in length (Matsuura and Sakagami 1973, Lee 2010).

*V. mandarinia* have a caste system made up of queens, workers, and males, each fulfilling duties integral to the success of the colony (Archer 1995). The life cycle begins with a solitary queen initiating nest foundation after overwintering in a self-excavated cavity in a soft ground-based substrate. Nest formation takes place over a number of weeks in the late spring. During this period, the queen builds and develops the nest, prepares to lay eggs, and feeds on arthropods and sap (Archer 1995). The colony begins in summer as the queen takes care of her brood and workers eventually begin to emerge. Once the queen has produced enough workers, the duties of the colony are transferred solely to the workers, while the queen remains in the confines of the nest and continues to lay eggs (Matsuura and Sakagami 1973, Archer 1995, Takahashi et al. 2004).

Mating season for *V. mandarinia* begins in early fall, with both new queens and reproductive males emerging (Matsuura and Sakagami 1973, Archer 1995). Males leave the nest before the queens to forage and to wait to mate with the newly emerging queens at the entrance of the nesting site (Matsuura 1984). The activity of the colony gradually decreases in the late fall before ceasing in the early winter, when queens need to find a site to overwinter (Archer 1995). Maturity from egg to adult is approximately 40 d (Matsuura 1984) and the colony cycle lasts approximately 6 mo, with the males and workers living for approximately 3 wk, while queens live as long as 12 mo when accounting for their overwintering period (Archer 1995).

The nests are assembled primarily in pre-existing ground-based cavities, such as burrows, snake holes, or rotting tree roots (Archer 1995). The nests can be fairly complex and vary in size and are made from foraged wood-based fibers. A larva matures in each cell (Matsurra and Yamane 1990). Archer (1995) reported a nest containing approximately 6,000 cells. Although the average nest can contain a few thousand separate cells, the actual colony size produced from those cells is usually much smaller. The variable cell count of each nest makes it difficult to estimate the size of the colony that will be produced from those cells. Despite this, Archer (1995) observed that a colony produced an average of approximately 200 males and 200 queens in a given cycle in addition to hundreds of workers.

*Vespa mandarinia* seems to be sensitive to high temperatures and prefers more temperate climates, areas of low elevation, and high amounts of precipitation for its nesting site (Kim et al. 2020, Zhu et al. 2020, Alaniz et al. 2021). However, there are reports of *V. mandarinia* attacking honey bee colonies at high altitudes, such as in the Himalayan ranges (Batra 1996). Furthermore, queens prefer ‘green’ environments, such as forested areas, parks, agricultural zones, and other herbaceous settings (Kim et al. 2020, Alaniz et al. 2021). This finding raises concerns about the risks to wild and cultivated bee populations that are in these environments. In addition, nest colonization within urban greenspaces has the potential to result in human conflicts with *V. mandarinia*. Liu et al. (2016) reported that in only a 4-mo period (July–October when the species is typically active), 42 people died and approximately 1,700 people were injured from suffering multiple stings in China’s Shaanxi Province.

Once *V. mandarinia* has occupied its new environment after initial nest colonization, it must feed and forage. Although the species is most known for its predation on social bees, it also feeds on sap from a number of different plant species that may lead to crop damage. Overwintering queens initially begin feeding on tree sap sources and some fruiting tree species. *Quercus* (oak) species were identified as an important sap source in which queens will begin to feed upon in mid-late April (Matsurra and Sakagami 1973, Matsurra 1984, Matsurra and Yamane 1990, Makino 2016). In addition, *V. mandarinia* has also been found to be a competitively dominant species among a number of major diurnal sap-feeding species (Yoshimoto and Nishidia 2009).

*Vespa mandarinia* aggressively preys on insect species. It preys on beetles, spiders, other social wasp species, but is most well-known for its mass attacks on honey bee species and their colonies (Matsuura 1984, Matsurra and Yamane 1990). *V. mandarinia*’*s* propensity to hunt honey bees has caused issues worldwide (Matsuura and Sakagami 1973), especially in instances where it has become established and local honey bee populations have not had the chance to adapt to its attacks.

**Fig. 1.**
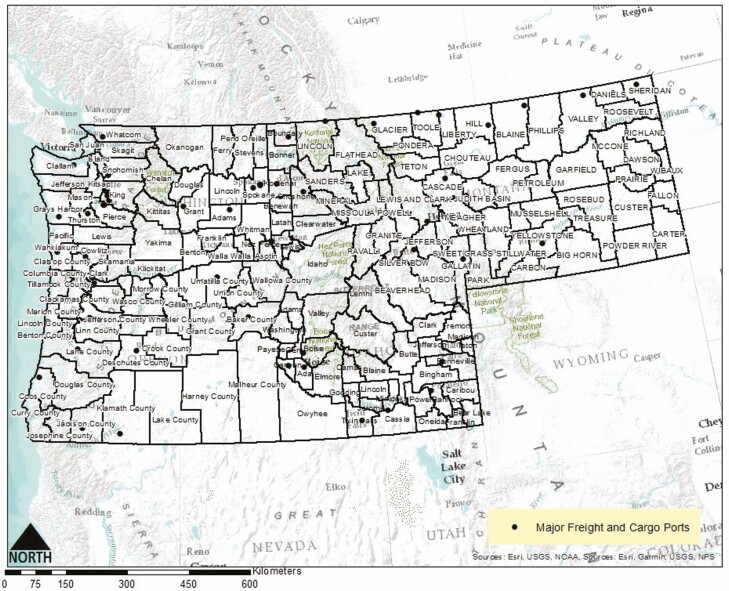
Major freight and cargo ports in the Pacific Northwest. The recent introduction of *Vespa mandarinia* in North America is thought to have resulted from economic trade activities between the United States and Asia.

Introduction of non-native species to new territories through natural dispersal such as flying or foraging is unlikely to occur over large geographic distances. However, unlike natural methods of non-native species introduction into new environments, human-mediated introduction is considered the leading cause of non-native biological invasions, not only in the United States, but also around the world (Vitousek et al. 1997). This is a result of extensive land transformations producing favorable conditions for invasion and accidental introductions due to international export and import commercial trade (Vitousek et al. 1997). Human-mediated introduction through economic trade is the likely reason *V. mandarinia* was accidentally introduced in northern Washington, considering the species was found close to the U.S./Canadian border near ports of entry and that the region serves as a destination for commercial trade commodities from Asia (Wilson et al. 2020) (Fig. 1). This is supported by the captures of *V. mandarinia* in both Vancouver, British Columbia and Washington that were found to originate from two separate lineages (Wilson et al. 2020). The individual captured in British Columbia had DNA from a lineage in Yamaguchi, Japan, whereas the captured specimen in Washington had DNA linked to a maternal lineage in Chungcheonuk-do, South Korea. Both these introductions in British Columbia and Washington were likely from separate mated queens, although the data could not ascertain whether these specimens were from the same populations or were introduced at the same time (Wilson et al. 2020). Despite this, the data seem to substantiate the role that human-mediated transport through economic trade has played in the introduction of *V. mandarinia* to North America and the Pacific Northwest.

Beyond global economic trade, cultural pursuits may serve as potential pathways for the introduction of *V. mandarinia*. The species is considered a delicacy in its endemic ranges in Asia and pupae and adults alike are consumed as food in multiple dishes and are often semidomesticated for these purposes (Mozhui et al. 2020). Obviously, specimens that are consumed no longer serve as a pest risk, but the transportation of the insect at any life stage where it is still living could inadvertently result in establishment if it were to escape and colonize a new nest site. Most ports of entry within the United States have safeguarding and inspection measures in place to prevent the importation of live insects and foreign species in cases like this. However, a 100% interception rate is highly unlikely, so the transportation of the insect for cultural purposes must be viewed as a viable potential pathway for its introduction to non-native regions of the United States.

### Effects Assessment

The effects assessment in our risk assessment assumes *V. mandarinia* queens survive their overwintering period and successfully initiate a new nest and establish a colony, potentially causing negative effects to environments in the Pacific Northwest. Those potential deleterious effects occur primarily through feeding and predation strategies of the colony, which result in crop damage and attacks on honey bee populations that can thereby impact pollination services.

There are distinct phases that *V. mandarinia* exhibit to attack a honey bee colony. The first phase begins with a solitary scout chemically marking a bee colony or hive by rubbing her terminal gastrite sternite directly on the targeted hive to signal to the rest of the colony of the availability of a source of food (Ono et al. 1995). Once the chemical pheromone has alerted other members of the colony, they will gather en masse and kill the adults in the hive (Matsuura 1984, Ono et al. 1995).

Honey bee species such as *Apis cerana japonica* that have coevolved with *V. mandarinia* in their native ranges have the ability to defend themselves against attack by alerting nestmates of incoming attack using chemical cues (Fujiwara et al. 2018, McClenaghan et al. 2019). They use a defense mechanism termed a ‘hot defensive bee ball’ in which hundreds of bees swarm a single hornet and generate enough heat and carbon dioxide around the attacker to kill it (Sugahara and Sakamoto 2009). Ono et al. (1995) observed through thermal imagery that the hot defensive bee ball was more than 47^o^C (116^o^F). Similarly, *A. c. japonica* has been documented to smear both plant-based materials and animal feces around hive entrances to disrupt attacks by *V. mandarinia* and the closely related species *Vespa soror* (Matilla et al. 2020).

Once a honey bee colony’s defenses are largely overcome, *V. mandarinia* begins its occupation phase and feeds on the colony’s brood for several days (Ono et al. 1995). For honey bee species that have not coevolved with *V. mandarinia*, such as *Apis mellifera,* which only have less effective stingers as a defense (Ugajin et al. 2012), complete annihilation of the colony is a likely outcome when attacked en masse by the hornet, termed the ‘slaughter’ phase (Matsuura and Sakagami 1973, Matsurra 1988). The slaughter phase involves mass attack in which the hornets can quickly dispatch an entire colony, mostly through decapitation using their mandibles. The slaughter event lasts between 1 and 6 hr and can result in the deaths of thousands of bees or entire colonies, in which the decapitated bees are often left in massive piles inside the hive (Matsuura 1984, Matsuura and Yamane 1990). The occupation and slaughter phase make the hornet a significant risk to vulnerable non-coevolved bee species. Should *V. mandarinia* become established in ecosystems outside its endemic range, wild bee colonies and apiaries may suffer extremely heavy losses resulting in substantial economic consequences to apiarists and the pollination services provided by wild bee and cultivated honey bees to hundreds of agricultural crops and plant species.

The European honey bee, *Apis mellifera,* may be at potential risk from *V. mandarinia* attack. It is a critically important pollinator around the world. In the United States, *A. mellifera* pollinates hundreds of crop species. Honey bees are the foremost insect pollinators and constitute an estimated economic benefit of nearly $12 billion or roughly 80% of the total pollination value in the United States (Choi and Kwon 2015).

The Pacific Northwest (primarily Washington and Oregon) is the nation’s leader in specialty crops including various varieties of fruits, nuts, and berries, with a total economic value of $4 billion annually (Houston et al. 2018). Considering that the majority of these crops are likely dependent on the pollination services provided by *A. mellifera*, the establishment and naturalization of *V. mandarinia* in the Pacific Northwest could pose high risks for agricultural producers. Beyond agricultural crop varieties, apiculture is also an agricultural sector at risk from the establishment *of V. mandarinia* in the Pacific Northwest. Furthermore, a recent survey of total honey bee colonies within the four states revealed that in June 2020, Washington State had an estimated 114,000 honey bee colonies, Oregon had an estimated 95,000, while Montana and Idaho had 110,000 and 107,000 colonies, respectively (USDA NASS 2021).

Beyond the potential risks *V. mandarinia* poses to agricultural and apicultural sectors, the insect also poses a risk to health and human safety. The U.S. Census Bureau estimated populations of more than 7.5 million residents in Washington, 4.2 million residents in Oregon, 1 million residents in Montana, and 1.7 million residents in Idaho in 2019 (U.S. Census Bureau 2019). Although it is statistically unlikely that even a small percentage of those populations would ever interact with *V. mandarinia*, the hornet kills dozens of people per year on average in Japan and causes sting-related injuries to thousands more (Dooley 2020).

### Exposure Assessment

The exposure assessment phase of a risk assessment involves drawing upon information from the stressor description and effects assessment and applies relevant data to the environment or ecosystems in question for the purpose of analysis to estimate the degree of risk, impacts, or potential consequences that the stressor may have in those environments. Accordingly, drawing from our stressor description and effects assessment of *V. mandarinia*, our exposure assessment analyzed the ecosystems and environments of the Pacific Northwest and compared them to the ecological requirements of the hornet.

Our analysis primarily focused on regions that match *V. mandarinia’s* climate and habitat suitability requirements and the presence and density of honey bee colonies. Suitable climate for *V. mandarinia* was based on minimum and maximum temperatures using plant hardiness zone maps of Washington, Oregon, Montana, and Idaho and comparing them to the climate in its native ranges in Asia because minimum and maximum temperatures have been cited as necessary abiotic factors critical to the establishment of viable insect populations (Zhu et al. 2020). Therefore, this serves as a good predictor of whether *V. mandarinia* queens would be able to survive their overwintering period.

The United States is divided into 13 separate plant hardiness zones across 10^o^F differences, which are based on minimum winter temperatures. These zones are further classified into two separate zones (A or B) by 5^o^F differences. Washington’s plant hardiness zones range from 4A (−34 to −31^o^C or −30 to −25^o^F) to 9A (−6 to −3.8^o^C or 20 to 25^o^F). Oregon shares similar zone ratings, which ranges from 4B (−31 to −28.9^o^C or −25 to −20^o^F) to 9A (−6 to −3.8^o^C or 20 to 25^o^F) (USDA Agricultural Research Service 2020). Montana’s and Idaho’s climate is generally much colder, ranging from 3A (−40 to −37.2°C or −40 to −35°F) to 6A (−23.3 to −20.6°C or −10 to −5°F) in Montana and 3B (−37.2 to −34.4°C or −35 to −40°F) to 7B (−15 to 12.2°C or 5 to 10°F) in Idaho.

*Vespa mandarinia’s* native ranges in Eastern and Southeast Asia include plant hardiness zones of 6A–13B (Magarey et al. 2008). However, without thorough and up-to-date distribution data of the insect within its endemic ranges or those regions within the Pacific Northwest, it is difficult to pinpoint the precise plant hardiness zones that the insect favor, resulting in what is most likely a highly generalized estimate (Magarey et al. 2008).

**Fig. 2.**
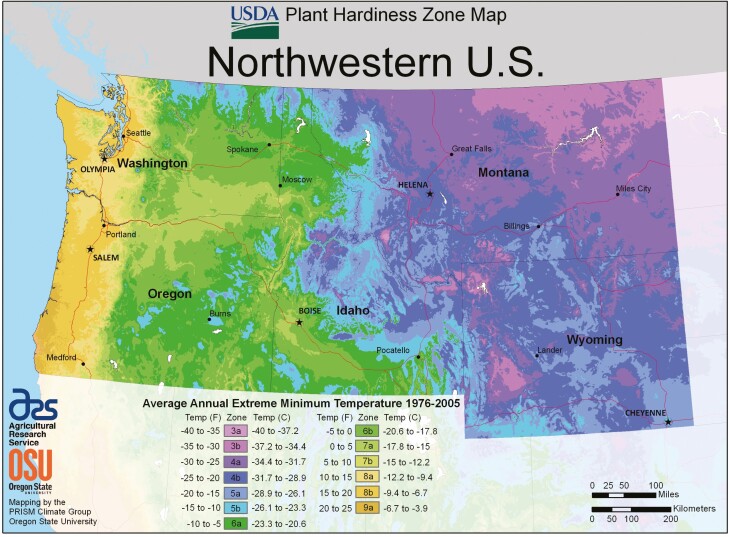
Plant hardiness zone (PHZ) map of the U.S. Pacific Northwest. PHZs are based on the average minimum winter temperature across a 30-yr time frame for a region and are used to help growers determine which plants may grow best depending on the zone they inhabit. This risk assessment used PHZs to determine suitable climate where *Vespa mandarinia* may overwinter. Washington and Oregon PHZs share some of the same PHZs that are present in *V. mandarinia’*s native ranges. These zones include 6A–9A, which include the majority of Washington and Oregon, indicating suitable climate for *V*. *mandarinia* to overwinter in and predate on honey bee populations. Montana and Idaho only share two of the same PHZs that are present in *V. mandarinia’*s native ranges. These zones are 6A–7B, found in northwestern Montana and much of northern and southwestern Idaho. (Source: USDA Agricultural Research Service 2020).

Consequently, there is overlap in plant hardiness zones between *V. mandarinia’s* natural range and areas in Washington, Oregon, Montana, and Idaho. For the purpose of the analysis, counties which contained PHZ’s below 6A were classified as ‘low’ risk. Counties with PHZ’s ranging from 6A to 7B were classified as ‘medium’ risk and counties with PHZ’s of 7B or greater were classified as ‘high’ risk. This overlap is primarily in the western and coastal regions of each state, but also in some inland regions as well (Fig. 2). Thus, there is risk that queens may be able to survive their overwintering period within these regions.

**Fig. 3.**
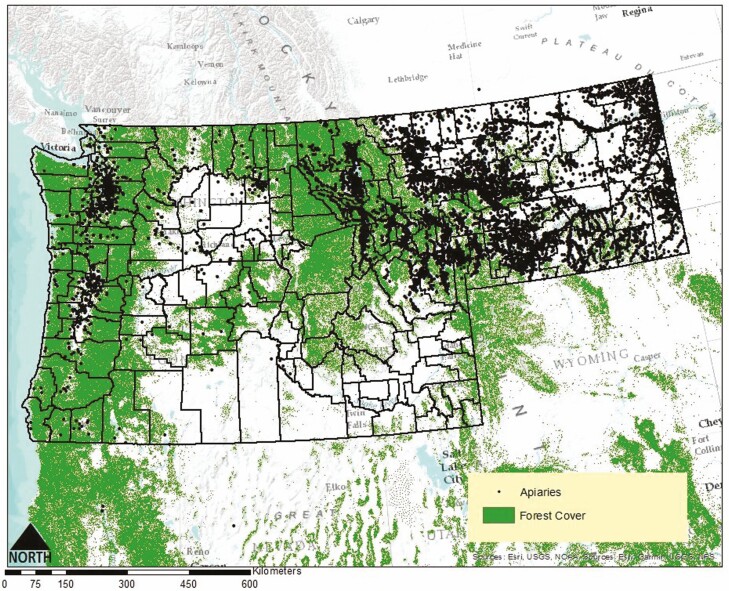
Forest cover in the Pacific Northwest with apiary distribution in Washington, Oregon, and Montana. Due to the Idaho Public Record Law, we were not permitted to obtain or display location of registered apiaries in the state. *Vespa mandarinia* prefers to colonize nests in ‘greenspaces.’ Considering the density of forest cover, particularly in the western portions of Washington, Oregon, Montana, and Idaho, these regions may serve as suitable habitat for nest establishment. Note the apiary distribution in relation to areas of dense forest cover.

The Pacific Northwest also has very dense forest cover. Blackard et al. (2008) estimated that the Pacific Northwest contained the highest densities of forest biomass in the contiguous United States, with an estimated 22 million acres of forested landcover in Washington and 32 million acres of forested landcover for Oregon. Montana has 25 million acres of forested land cover, while Idaho has 21 million acres (USDA 2017). Considering that *V. mandarinia* prefers to establish and colonize nests within green, herbaceous environments, the Pacific Northwest serves as a suitable region within the United States for it to establish and proliferate (Fig. 3). To assign forest cover into categories of ‘low,’ ‘medium,’ or ‘high,’ we used a forest cover dataset that classified forest cover in the northwestern United States by percentage. Counties that contained forest cover 0–33% were classified as ‘low,’ counties with forest cover estimates 33–66% were classified as ‘medium,’ and counties estimated to contain forest cover estimates 66–99% were classified as ‘high.’

We obtained data on honey bee colony densities and distribution by county for Washington, Oregon, Montana, and Idaho based on registered apiaries and number of individual hives of each apiary. The information was then summed for each county for a total number of individual hives in each state, with percentage for each county relative to the total across the four states.

For Washington, the results showed that Grant County, Yakima County, and Skagit County comprised the majority of honey bee colonies at 40.2%, 12.9%, and 11.0%, respectively, accounting for 64% of the state’s apicultural populations. The remaining 36% of apiary populations among Washington’s counties ranged from 0.01 to 3.5% of the state’s total apiary populations (Table 1). For Oregon, the results indicated that Malheur County, Linn County, Yamhill County, Clackamas County, and Marion County accounted for approximately 75% of the state’s apicultural populations (Table 1).

**Table 1.** Total honey bee hives by county in Washington State and Oregon

Washington counties	Total hives and % relative to state total	Oregon counties	Total hives and % relative to state total
Adams	100 (0.1%)	Baker	0 (0.0%)
Asotin	263 (0.3%)	Benton	4,599 (6.0%)
Benton	42 (0.0%)	Clackamas	8,026 (10.5%)
Chelan	527 (0.6%)	Clatsop	30 (0.0%)
Clallam	167 (0.2%)	Columbia	7 (0.0%)
Clark	2,234 (2.5%)	Coos	143 (0.2%)
Columbia	1,157 (1.3%)	Crook	29 (0.0%)
Cowlitz	201 (0.2%)	Curry	18 (0.0%)
Douglas	3,015 (3.3%)	Deschutes	23 (0.0%)
Ferry	27 (0.0%)	Douglas	432 (0.6%)
Franklin	3,009 (3.3%)	Gilliam	0 (0.0%)
Garfield	508 (0.6%)	Grant	686 (0.9%)
Grant	36,520 (40.2%)	Harney	20 (0.0%)
Grays Harbor	94 (0.1%)	Hood River	2,600 (3.4%)
Island	467 (0.5%)	Jackson	350 (0.5%)
Jefferson	194 (0.2%)	Jefferson	12 (0.0%)
King	2,935 (3.2%)	Josephine	26 (0.0%)
Kitsap	485 (0.5%)	Klamath	40 (0.1%)
Kittitas	203 (0.2%)	Lake	15 (0.0%)
Klickitat	29 (0.0%)	Lane	742 (1.0%)
Lewis	605 (0.7%)	Lincoln	59 (0.1%)
Lincoln	111 (0.1%)	Linn	12,457 (16.4%)
Mason	64 (0.1%)	Malheur	19,100 (25.1%)
Okanogan	544 (0.6%)	Marion	7,832 (10.3%)
Pacific	37 (0.0%)	Morrow	0 (0.0%)
Pend Oreille	17 (0.0%)	Multnomah	1,230 (1.6%)
Pierce	785 (0.9%)	Polk	2,543 (3.3%)
San Juan	95 (0.1%)	Sherman	0 (0.0%)
Skagit	10,020 (11.0%)	Tillamook	112 (0.1%)
Skamania	101 (0.1%)	Umatilla	3,195 (4.2%)
Snohomish	2,898 (3.2%)	Union	25 (0.0%)
Spokane	3,623 (4.0%)	Wallowa	0 (0.0%)
Stevens	3,169 (3.5%)	Wasco	140 (0.2%)
Thurston	1,305 (1.4%)	Washington	1,174 (1.5%)
Wahkiakum	36 (0.0%)	Wheeler	1,000 (1.3%)
Walla Walla	1,915 (2.1%)	Yamhill	9,767 (12.8%)
Whatcom	1,533 (1.7%)		
Whitman	25 (0.0%)		
Yakima	11,675 (12.9%)		

Note the high proportions of honey bee hives in Grant, Skagit, and Yakima counties relative to the rest of Washington state. For Oregon, note Malheur, Linn, Clackamas, Marion, and Yamhill counties relative to the rest of the state. Additionally, these counties also fall within the plant hardiness zones identified to provide suitable climate where *Vespa mandarinia* may overwinter. See Figs. 2 and 3 (Freedom of Information Act Request from Washington Department of Agriculture and Oregon Department of Agriculture).

For Montana, the results showed that Richland, Lewis and Clark, and Fergus counties made up approximately 20% of apiaries in the state (Table 2). Canyon, Elmore, Gooding, Owyhee, Ada, and Payette counties accounted for roughly 42% of apiaries among Idaho’s 44 counties (Table 2). The distribution of apiaries in Washington, Oregon, and Montana is displayed graphically (Fig. 3). Per the Idaho Public Records Law, we were not permitted to obtain or display locational information of registered apiaries in the state. Apiaries were classified into categories of ‘low,’ ‘medium,’ or ‘high’ densities based on a county’s total apiaries relative to the total number of apiaries in a given state. Counties that contained an apiary percentage of 0–2% were classified as ‘low,’ counties with an apiary percentage of 3–4% were classified as ‘medium,’ and counties with an apiary percentage of 5% and above were classified as ‘high.’ All percentages were rounded to the nearest whole number for the purpose of classification system.

**Table 2.** Total honey bee hives by county in Montana and Idaho

Montana counties	Total hives and % relative to state total	Idaho counties	Total hives and % relative to state total
Beaverhead	3,349 (1.3%)	Ada	5,781 (4.7%)
Big Horn	6,911 (2.6%)	Adams	1,320 (1.0%)
Blaine	5,316 (2.0%)	Bannock	3,679 (3.0%)
Broadwater	3,353 (1.3%)	Bear Lake	2,854 (2.3%)
Carbon	4,509 (1.7%)	Benewah	200 (0.1%)
Carter	3,203 (1.2%)	Bingham	3,264 (2.6%)
Cascade	8,901 (3.4%)	Blaine	1,460 (1.2%)
Choteau	8,841 (3.4%)	Boise	3,285 (2.7%)
Custer	2,882 (1.1%)	Bonner	748 (0.6%)
Daniels	3,260 (1.2%)	Bonneville	3,358 (2.7%)
Dawson	4,129 (1.6%)	Boundary	1,998 (1.6%)
Deer Lodge	916 (0.3%)	Butte	1,661 (1.3%)
Fallon	4,868 (1.8%)	Camas	800 (0.6%)
Fergus	12,000 (4.6%)	Canyon	10,866 (8.9%)
Flathead	5,486 (2.1%)	Caribou	2,843 (2.3%)
Gallatin	5,500 (2.1%)	Cassias	2,568 (2.1%)
Garfield	4,572 (1.7%)	Clark	750 (0.6%)
Glacier	4,104 (1.5%)	Clearwater	802 (0.6%)
Golden Valley	2,919 (1.1%)	Custer	1,515 (1.2%)
Granite	1,666 (0.6%)	Elmore	7,165 (5.9%)
Hill	1,471 (0.5%)	Franklin	3,934 (3.2%)
Jefferson	1,583 (0.6%)	Fremont	1,854 (1.5%)
Judith Basin	7,581 (2.9%)	Gem	3,306 (2.7%)
Lake	5,612 (2.1%)	Gooding	7,157 (5.9%)
Lewis and Clark	15,240 (5.9%)	Idaho	2,451 (2.0%)
Liberty	742 (0.2%)	Jefferson	1,871 (1.5%)
Lincoln	1,093 (0.4%)	Jerome	1,916 (1.5%)
Madison	5,641 (2.1%)	Kootenai	1,882 (1.5%)
McCone	916 (0.3%)	Latah	1,166 (0.9%)
Meagher	3,486 (1.3%)	Lemhi	1,164 (0.9%)
Mineral	1,171 (0.4%)	Lewis	945 (0.7%)
Missoula	3,801 (1.4%)	Lincoln	1,970 (1.6%)
Musselshell	3,171 (1.2%)	Madison	2,651 (2.1%)
Park	6,554 (2.5%)	Minidoka	1,211 (0.9%)
Petroleum	2,186 (0.8%)	Nez Perce	2,100 (1.7%)
Phillips	4,079 (1.5%)	Oneida	3,306 (2.7%)
Pondera	4,826 (1.8%)	Owyhee	7,587 (6.2%)
Powder River	3,958 (1.5%)	Payette	5,478 (4.5%)
Powell	2,152 (0.8%)	Power	2,974 (2.4%)
Prairie	1,370 (0.5%)	Shoshone	52 (0.0%)
Ravalli	7,364 (3.0%)	Teton	1,250 (1.0%)
Richland	21,723 (8.4%)	Twin Falls	2,756 (2.2%)
Roosevelt	3,291 (1.2%)	Valley	1,060 (0.8%)
Rosebud	4,379 (1.6%)	Washington	4,317 (3.5%)
Sanders	6,215 (2.4%)		
Sheridan	4,673 (1.8%)		
Silver Bow	906 (0.3%)		
Stillwater	6,733 (2.6%)		
Sweet Grass	4,817 (1.8%)		
Teton	5,058 (1.9%)		
Toole	1,227 (0.4%)		
Treasure	1,726 (0.6%)		
Valley	3,030 (1.1%)		
Wheatland	2,889 (1.1%)		
Wibaux	1,832 (0.7%)		
Yellowstone	8,421 (3.2%)		

Note the high proportions of honey bee hives in Richland, Lewis and Clark, and Fergus counties relative to the rest of Montana state. For Idaho, note Canyon, Elmore, Gooding, Owyhee, Payette, and Ada counties relative to the rest of the state. Additionally, the counties in Idaho also fall within the plant hardiness zones identified to provide suitable climate where *Vespa mandarinia* may overwinter. See Figs. 2 and 3 (Freedom of Information Act Request from Montana Department of Agriculture and Idaho State Department of Agriculture).

Lastly, we examined major freight hubs and cargo ports in the Pacific Northwest by county on the basis that economic trade is historically responsible for numerous accidental invasive species introductions. If a county contained more than one major port or freight hub, that county received a risk rating of ‘3.’ If a county contained one major port or freight hub, it received a risk rating score of ‘2.’ If a county did not contain a major port or freight hub, it received a risk rating of ‘1’ (Fig. 1).

Based on the apicultural distribution and the plant hardiness zone maps of the four states and the previously stated habitat suitability for overwintering, the majority of Washington’s and Oregon’s counties fall within these suitable temperature ranges, with the exception of northern Okanogan, Ferry, Stevens, Pend Oreille, and portions of Wallowa, Baker, Grant, Harney, Lake, Malheur, Crook, Deschutes, and Klamath counties (Fig. 2). In Idaho, the majority of the southern and western counties match these suitable temperature ranges (Fig. 2). In contrast, only parts of Sanders, Mineral, and Lake Counties in Montana provide temperature ranges which match *V. mandarinia*’s endemic environments (Fig. 2).

Although the plant hardiness zones are likely an overgeneralization of suitable habitat for *V. mandarinia*, it is nonetheless concerning that a large portion of Washington’s, Oregon’s, and Idaho’s apicultural industry lies within these zones of potentially suitable climate and proximity to areas of suitable habitat for nest colonization (Figs. 2 and 3).

### Risk Characterization and Discussion

To estimate the risk *V. mandarinia* poses to Washington, Oregon, Montana, and Idaho, we used a risk rating and scoring system based on an approach used by Schleier et al. (2008) to rank the relative risk of the following categories and criteria: (1) climate suitability for *V. mandarinia* to overwinter based on plant hardiness zones (ideal plant hardiness zone score in Tables 3–6), (2) habitat suitability to colonize nests in ‘green’ environments, which was based on dense forest cover in the Pacific Northwest, (3) density of apiaries by county, and (4) the proximity of introduction pathways (major port or freight hubs) that may increase the risk of establishment.

**Table 3.** Risk rating table for *Vespa mandarinia* establishment in Washington State

County	Ideal plant hardiness zone score	Apiary density score	Dense forest biomass score	Proximity to introduction pathway score	Overall risk rating score (ORS)
Adams	2	1	1	2	6
Asotin	2	1	1	2	6
Benton	2	1	1	3	7
Chelan	2	1	3	1	7
Clallam	3	1	3	2	9
Clark	3	2	3	2	10
Columbia	2	2	2	2	8
Cowlitz	3	1	2	3	9
Douglas	2	2	1	1	6
Ferry	1	1	2	1	5
Franklin	2	2	1	3	8
Garfield	2	1	1	1	5
Grant	2	3	1	2	8
Grays Harbor	3	1	3	2	9
Island	3	1	2	1	7
Jefferson	3	1	3	1	8
King	3	2	2	3	10
Kitsap	3	1	2	1	7
Kittitas	2	1	2	1	6
Klickitat	2	1	1	1	5
Lewis	3	1	3	1	8
Lincoln	2	1	1	1	5
Mason	3	1	3	1	8
Okanogan	1	1	1	1	4
Pacific	3	1	3	1	8
Pend Oreille	1	1	2	1	5
Pierce	3	1	3	3	10
San Juan	3	1	3	1	8
Skagit	2	3	3	2	10
Skamania	2	1	3	1	7
Snohomish	3	2	3	3	11
Spokane	2	2	1	3	8
Stevens	2	2	2	1	7
Thurston	3	2	2	1	8
Wahkiakum	3	1	3	1	8
Walla Walla	2	2	1	2	7
Whatcom	2	2	3	2	9
Whitman	2	1	1	1	5
Yakima	2	3	2	1	8

An overall risk rating score (ORS) of 1–4 equals low risk. An ORS of 5–8 equals medium risk and an ORS of 9–12 equals high risk. Low ORS is highlighted in green, medium in yellow, and high in red.

**Table 4.** Risk rating table for *Vespa mandarinia* establishment in Oregon

Oregon counties	Ideal plant hardiness zone score	Apiary density score	Dense forest biomass score	Proximity to introduction pathway score	Overall risk rating score (ORS)
Baker	1	1	1	2	5
Benton	3	3	2	2	10
Clackamas	3	3	2	1	9
Clatsop	3	1	3	2	9
Columbia	3	1	3	1	8
Coos	3	1	3	2	8
Crook	1	1	1	1	4
Curry	3	1	3	1	8
Deschutes	1	1	1	2	5
Douglas	3	2	3	2	10
Gilliam	2	1	1	1	5
Grant	1	2	1	1	5
Harney	1	1	1	1	4
Hood River	3	2	2	2	9
Jackson	3	1	2	2	8
Jefferson	2	1	1	1	5
Josephine	3	1	3	1	8
Klamath	1	1	2	2	6
Lake	1	1	1	1	4
Lane	3	1	3	2	9
Lincoln	3	1	3	2	9
Linn	3	3	2	2	10
Malheur	1	3	1	1	6
Marion	3	3	2	2	10
Morrow	2	1	1	1	5
Multnomah	3	2	1	3	9
Polk	3	2	3	2	10
Sherman	2	1	1	1	5
Tillamook	3	1	3	1	8
Umatilla	2	2	1	2	7
Union	2	1	1	2	6
Wallowa	1	1	1	1	4
Wasco	2	1	1	1	5
Washington	3	1	1	2	7
Wheeler	2	1	1	1	5
Yamhill	3	3	2	1	9

An overall risk rating score (ORS) of 1–4 equals low risk. An ORS of 5–8 equals medium risk and an ORS of 9–12 equals high risk. Low ORS is highlighted in green, medium in yellow, and high in red.

**Table 5.** Risk rating table for *Vespa mandarinia* establishment in Montana

County	Ideal plant hardiness zone score	Apiary density score	Dense forest biomass score	Proximity to introduction pathway score	Overall risk rating score (ORS)
Beaverhead	1	1	2	1	5
Big Horn	1	1	1	1	4
Blaine	1	1	1	2	5
Broadwater	1	1	2	1	5
Carbon	1	1	1	1	4
Carter	1	1	1	2	5
Cascade	1	2	1	2	6
Choteau	1	2	1	1	5
Custer	1	1	1	1	4
Daniels	1	1	1	2	5
Dawson	1	1	1	1	4
Deer Lodge	1	1	2	1	5
Fallon	1	1	1	1	4
Fergus	1	3	1	2	7
Flathead	1	1	3	2	7
Gallatin	1	1	2	2	6
Garfield	1	1	1	1	4
Glacier	1	1	2	2	6
Golden Valley	1	1	1	1	4
Granite	1	1	3	1	6
Hill	1	1	1	2	5
Jefferson	1	1	3	1	6
Judith Basin	1	2	2	1	6
Lake	2	1	2	1	6
Lewis & Clark	1	3	3	2	9
Liberty	1	1	1	2	5
Lincoln	1	1	3	2	7
Madison	1	1	2	1	5
McCone	1	1	1	1	4
Meagher	1	1	2	1	5
Mineral	2	1	3	1	7
Missoula	1	1	3	2	7
Musselshell	1	1	2	1	5
Park	1	2	2	1	6
Petroleum	1	1	1	1	4
Phillips	1	1	1	1	4
Pondera	1	1	2	1	5
Powder River	1	1	2	1	5
Powell	1	1	3	1	7
Prairie	1	1	1	1	4
Ravalli	1	2	3	1	7
Richland	1	3	1	1	6
Roosevelt	1	1	1	1	4
Rosebud	1	1	1	1	4
Sanders	2	1	3	1	7
Sheridan	1	1	1	2	5
Silver Bow	1	1	3	2	7
Stillwater	1	2	1	1	5
Sweet Grass	1	1	1	1	4
Teton	1	1	2	1	5
Toole	1	1	1	2	5
Treasure	1	1	1	1	4
Valley	1	1	1	2	5
Wheatland	1	1	1	1	4
Wibaux	1	1	1	1	4
Yellowstone	1	2	1	2	6

An overall risk rating score (ORS) of 1–4 equals low risk. An ORS of 5–8 equals medium risk and an ORS of 9–12 equals high risk. Low ORS is highlighted in green, medium in yellow, and high in red.

**Table 6.** Risk rating table for *Vespa mandarinia* establishment in Idaho

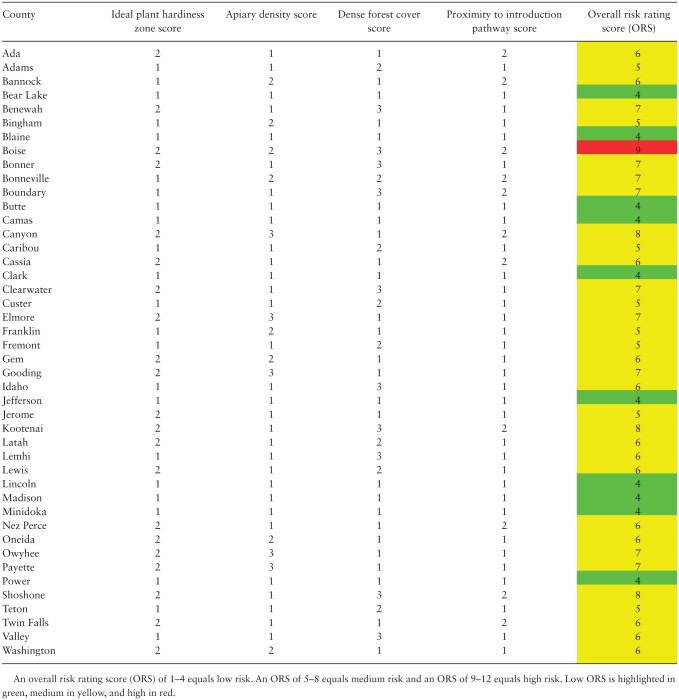

Those counties in each of the four states with high plant hardiness zone designations received a score of ‘3.’ Counties with medium to low plant hardiness zone designations received a risk rating of ‘2’ or ‘1,’ respectively. Similarly, counties with high densities of forest cover received a risk rating score of ‘3’ and counties with medium to low forest biomass received risk rating scores of ‘2’ and ‘1,’ respectively. Counties containing high totals of honey bee hives received a risk rating of ‘3,’ while those containing low numbers of honey bee hives relative to the rest of the state received risk ratings of ‘2’ and ‘1.’ Lastly, the introduction pathway score was based on major port and freight hubs contained in counties of the Pacific Northwest. If a county contained more than one major port or freight hub, that county received a risk rating of ‘3.’ If a county contained one major port or freight hub, it received a risk rating score of ‘2.’ If a county did not contain a major port or freight hub, it received a risk rating of ‘1.’

**Fig. 4.**
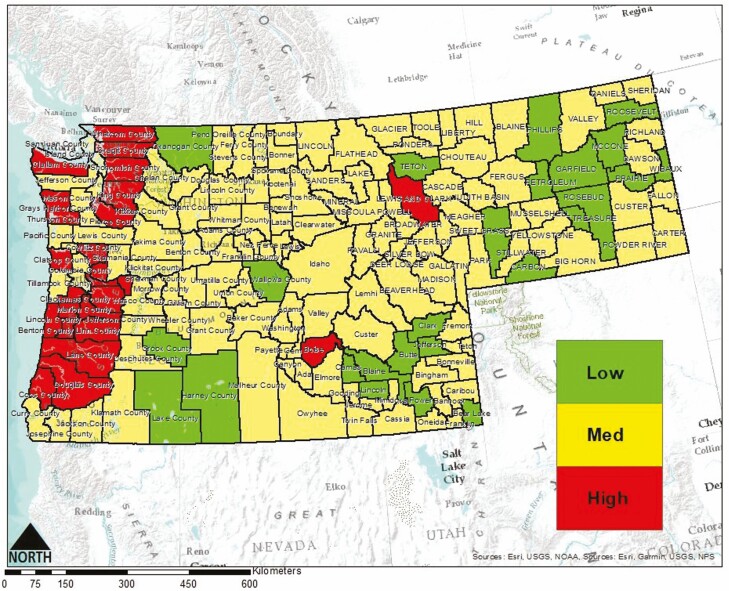
County-by-county color-coded risk map of *Vespa mandarinia* establishment in the Pacific Northwest.

The scores were then summed across each risk factor for each county for a total possible overall risk score (ORS) of 12. Those counties which received an ORS of 1–4 received a ‘low’ risk rating, while counties that received an ORS of 5–8 or 9–12 received a risk rating of ‘medium’ or ‘high,’ respectively (Tables 3–6). These results are also shown visually (Fig. 4). Each factor was weighted equally and it is important to note that this combined with ranking the risk of a particular county as either low, medium, or high introduces considerable model and parameter uncertainty that may overestimate risk (discussed in more detail below).

Our results identified 23 counties at a high risk of establishment. Washington contained 9 high-risk counties, while Oregon contained 12. For Washington, these counties were Clallam, Clark, Cowlitz, Grays Harbor, King, Pierce, Skagit, Snohomish, and Whatcom. The high-risk Oregon counties were Benton, Clackamas, Clatsop, Douglas, Hood River, Lane, Lincoln, Linn, Marion, Multnomah, Polk, and Yamhill. Of the remaining 54 counties in Washington and Oregon, 49 were found to be at a medium risk of establishment, while only 5 were found to be at a low risk of establishment by *V. mandarinia*. Montana and Idaho contained one high-risk county each: Lewis and Clark in Montana and Boise in Idaho. Additionally, 17 Montana counties were low risk, while Idaho contained 10 low-risk counties. The remaining 72 counties in the two states were estimated to be at medium risk.

The results of the establishment risk assessment of *V. mandarinia* in the Pacific Northwest suggest that the establishment may present serious economic consequences for the region, especially for apiculture and crop-pollinated agriculture sectors. Federal and state regulatory agencies, as well as apicultural and agricultural industries should therefore take immediate action to develop plans and methodologies to prevent further naturalization of *V. mandarinia* in Washington, Oregon, Montana, and Idaho, with the ultimate goal of complete eradication of the species.

#### Uncertainty Analysis

Our risk assessment had the goal of delineating the biological requirements of *V. mandarinia* and analyzing its known ecological relationships with the environments of the Pacific Northwest to estimate the establishment risk on a county-by-county basis. The results suggest a number of potential high, medium, and low risk factors that may aid in its establishment in the Pacific Northwest and thereby help key personnel focus on areas for surveillance.

However, our risk assessment used a tier-1 or screening-level approach with additive risk factors and equal weights, which is usually employed when there is a lack of quantitative or spatial data. Considering that *V. mandarinia* was recently introduced into the Pacific Northwest, there are few data concerning the species’ current distribution within Washington or elsewhere in the Pacific Northwest. Moreover, there is very little published literature on the species, with most of the published research dating back to the 1970s through the 1990s. Furthermore, although there has been somewhat of a recent resurgence in the literature on the species due to the recent introduction of the species into North America, most of this research also relied heavily on the same aforementioned research that was published decades ago.

With this scarcity of data and lack of knowledge concerning the species’ ecological relationships and distribution in the Pacific Northwest, our risk assessment relied on only a few of the biotic and abiotic requirements that may sustain or hamper establishment success of the hornet. For example, the use of plant hardiness zones to delineate habitat suitability for *V. mandarinia* in the Pacific Northwest likely considerably overestimates the areas in which the species could survive its overwintering period and establish new colonies the following year. Instead of using plant hardiness zones, other approaches may be more accurate and, therefore, informative. For example, CLIMEX or Maxent species distribution modeling may prove useful as more worldwide occurrence data and documented physiological tolerances become available (Kriticos et al. 2015, Kumar et al. 2016, Zhu et al. 2020). Accordingly, more detailed research regarding the ecological relationships and life cycle of *V. mandarinia* in the Pacific Northwest needs to be undertaken to form a more complete picture of what can actually be defined as suitable habitat within this region.

Our inclusion of major port and freight hubs and honey bee hive density arguably is not as important as the climate and habitat suitability factors (Zhu et al. 2020). In addition, by including port and freight hubs, our establishment risk assessment incorporates elements that might be more relevant to an introduction risk assessment. However, we believe including port and freight hubs is important because of examples such as Japanese beetle, *Popillia japonica* Newman. In Montana, the Japanese beetle was accidentally introduced at the Billings airport because of airplane transport and has only established in a relatively small area surrounding it (Montana Department of Agriculture 2014). Consequently, we believe port hubs can be important factors not only in introduction, but also establishment.

Data on honey bee hive distribution and density were based solely on hives that were managed by registered apiaries and did not take into account hives that may be managed by unregistered beekeepers. In addition, although the risk to apiaries may be easier to estimate, our risk assessment was not able to assess the risk to the pollination services provided by wild bee populations considering that to our knowledge no data exist on estimates of wild bee populations in the Pacific Northwest.

In addition, risk rating systems in qualitative or semiquantitative risk assessments often lack the confidence to accurately discern between quantitatively small and quantitatively large risks. This can result in errors such as the assignment of higher risk ratings to either a particular, or multiple, risk situations, which may in reality actually differ quantitatively by orders of magnitude (Cox et al. 2005). The risk rating system also introduces model uncertainty with the four additive risk factors being weighted equally and the assignment of low, medium, or high risk classification introduces parameter uncertainty. Combined, this produces outcomes that may considerably overestimate risk in a given county.

Furthermore, our risk characterization’s reliance on estimating risk on a county-by-county basis is at a coarse scale, which likely results in an over or under estimation of the actual risk in a given area. However, and to reiterate, our assessment is meant to be used by decision makers to identify counties in the region that are potentially at high risk of establishment. Future assessments should focus on estimating risk in the region at a finer scale.

## References

[CIT0001] Alaniz, A. J., M. A. Carvajal, and P. M. Vergara. 2021. Giants are coming? Predicting the potential spread and impacts of the giant Asian hornet (*Vespa mandarinia*, Hymenoptera:Vespidae) in the USA. Pest Manag. Sci. 77: 104–112. 32841491 10.1002/ps.6063

[CIT0002] Archer, M. E . 1995. Taxonomy, distribution, and nesting biology of the *Vespa mandarinia* group (Hym., Vespinae). Entomol. Mon. Mag. 131: 47–53.

[CIT0004] Batra, S. W. T . 1996. Biology of Apis Laboriosa Smith, a pollinator of apples at high altitude in the Great Himalaya Range of Garhwal, India, (Hymenoptera: Apidae). J. Kans. Entomol. Soc. 69:177–181.

[CIT0005] Blackard, J. A., M. V. Finco, E. H. Helmer, G. R. Holden, M. L. Hoppus, D. M. Jacobs, A. J. Lister, G. G. Moisen, M. D. Nelson, R. Riemann, et al. 2008. Mapping U.S. forest biomass using nationwide forest inventory data and moderate resolution information. Remote. Sens. Environ. 112:1658–1677.

[CIT0006] Choi, M. B., and O. Kwon. 2015. Occurrence of Hymenoptera (wasps and bees) and their foraging in the southwestern part of Jirisan National Park, South Korea. J. Ecol. Environ. 38:367–374.

[CIT0007] Cox, L. A., Jr, D. Babayev, and W. Huber. 2005. Some limitations of qualitative risk rating systems. Risk. Anal. 25: 651–662. 16022697 10.1111/j.1539-6924.2005.00615.x

[CIT0008] Dooley, B . 2020. In Japan, the ‘Murder Hornet’ Is Both a Lethal Threat and a Tasty Treat. The New York Times, 20 July 2020. Available from https://www.nytimes.com/2020/05/05/world/asia/murder-hornet-japan.html.

[CIT0009] EPA. 1998. Guidelines for Ecological Risk Assessment. EPA/630/R-95/002F. April 1998. U.S. Federal Register 63(93):26846–26924. U.S. Environmental Protection Agency, Washington, DC. 188 pages.

[CIT0010] Fujiwara, A., M. Sasaki, and I. Washitani. 2018. First report on the emergency dance *of Apis cerana japonica*, which induces odorous plant material collection in response to *Vespa mandarinia japonica* scouting. Entomol. Sci. 21:93–96.

[CIT0011] Herms, D. A., and D. G. McCullough. 2014. Emerald ash borer invasion of North America: history, biology, ecology, impacts, and management. Annu. Rev. Entomol. 59: 13–30. 24112110 10.1146/annurev-ento-011613-162051

[CIT0012] Houston, L., S. Capalbo, C. Seavert, M. Dalton, D. Bryla, and R. Sagili. 2018. Specialty fruit production in the Pacific Northwest: adaptation strategies for a changing climate. Clim. Change. 146:1159–1171.

[CIT0013] Kim, W. M., S. Y. Kim, and W. Song. 2020. Microhabitat characteristics affecting the occurrence and diversity of queen hornets (Genus *Vespa*) in an urban green Area. Landsc. Ecol. Eng. 16:173–186.

[CIT0014] Kriticos, D., G. Mawald, T. Yonow, E. Zurcher, N. Herrmann, and R. Sutherst. 2015. Climex Version 4: exploring the effects of climate on plants, animals and diseases. CSIRO, Canberra, Australia.

[CIT0015] Kumar, S., W. L. Yee, and L. G. Neven. 2016. Mapping global potential risk of establishment of *Rhagoletis pomonella* (Diptera: Tephritidae) using MaxEnt and CLIMEX niche models. J. Econ. Entomol. 109: 2043–2053. 27452001 10.1093/jee/tow166

[CIT0016] Lee, J. X. Q . 2010. Notes on *Vespa analis and Vespa mandarinia* (Hymenoptera: *Vespidae* in Hong Kong, and a key to all Vespa species known from the SAR. Hong Kong Entomol. Bull. 2:31–36.

[CIT0017] Liu, Z., X. D. Li, B. H. Guo, Y. Li, M. Zhao, H. Y. Shen, Y. Zhai, X. L. Wang, and T. Liu. 2016. Acute interstitial nephritis, toxic hepatitis and toxic myocarditis following multiple Asian giant hornet stings in Shaanxi Province, China. Environ. Health. Prev. Med. 21: 231–236. 26910407 10.1007/s12199-016-0516-4 PMC4907929

[CIT0018] Magarey, R. D., D. M. Borchert, and J. W. Schlegel. 2008. Global plant hardiness zones for phytosanitary risk analysis. Sci. Agric. 65:54–59.

[CIT0019] Makino, S . 2016. Post-hibernation ovary development in queens of the Japanese giant hornet *Vespa mandarinia* (Hymenoptera: Vespidae). Entomol. Sci. 19:440–443.

[CIT0020] Matilla, H., G. Otis, L. Nguyen, H. Pham, O. Knight, and N. Phan. 2020. Honey bee (*Apis cerana*) use animal feces as a tool to defend colonies against group attack by giant hornets (*Vespa soror*). PLoS ONE. 15:1–24. 10.1371/journal.pone.0242668 PMC7725375 33296376

[CIT0021] Matsuura, M . 1984. Comparative biology of the five Japanese species of the genus *Vespa* (Hymenoptera, Vespidae). Bull. Faculty. Agric. 69:1–131.

[CIT0022] Matsurra, M . 1988. Ecological study on Vespine wasps (Hymenoptera: Vespidae) attacking honey bee colonies. Seasonal changes in the frequency of visits to apiaries by Vespine wasps and damage inflicted, especially in the absence of artificial protection. Appl. Entomol. Zool. 23:428–440.

[CIT0023] Matsuura, M., and S. F. Sakagami. 1973. A bionomic sketch of the giant hornet, *Vespa mandarinia, a* serious pest for Japanese apiculture. J. Fac. Sci Hokaido Univ. (Zoology) 19:125–162.

[CIT0024] Matsurra, M., and S. Yamane. 1990. Biology of the vespine wasps. Springer-Verlag, Berlin, Germany, 1–167.

[CIT0025] McClenaghan, B., M. Schlaf, M. Geddes, J. Mazza, G. Pitman, K. McCallum, S. Rawluk, K. Hand, and G. W. Otis. 2019. Behavioral responses of honey bees, *Apis cerana* and *Apis mellifera*, to *Vespa mandarinia* marking and alarm pheromones. J. Apic. Res. 58:141–148.

[CIT0026] Montana Department of Agriculture. 2014. Cooperative agricultural pest survey results. Montana State Department of Agriculture, Helena, MT.

[CIT0027] Mozhui, L., L. N. Kakati, P. Kiewhuo, and S. Changkija. 2020. Traditional knowledge of the utilization of edible insects in Nagaland, North-East India. Foods 9:1–17. 10.3390/foods9070852 PMC7404660 32629940

[CIT0028] NRC. 1983. Risk assessment in the federal government: managing the process. National Research Council. National Academy Press, Washington, DC. 25032414

[CIT0029] NRC. 1996. Understanding risk: informing decisions in a democratic society. National Research Council. National Academy Press, Washington, DC.

[CIT0030] Ono, M., T. Igarashi, E. Ohno, and M. Sasaki. 1995. Unusual thermal defense by a honeybee against mass attack by hornets. Nature. 377:334–336.

[CIT0031] Schleier, J. J., S. E. Sing, and R. K. D. Peterson. 2008. Regional ecological risk assessment for the introduction of *Gambusia affinis* (western mosquitofish) into Montana watersheds. Biol. Invasions. 10:1277–1287.

[CIT0032] SETAC. 1994. Aquatic dialogue group: pesticide risk assessment and mitigation. Society for Environmental Toxicology and Chemistry and SETAC Foundation for Environmental Education, Pensacola, FL.

[CIT0033] Smith-Pardo, A. H., J. M. Carpenter, and L. Kimsey. 2020. The diversity of hornets in the genus *Vespa* (Hymenoptera: Vespidae; Vespinae), their importance and interceptions in the United States. Insect. Syst. Diver. 4:1–27.

[CIT0034] Soliman, T., M. C. M. Mourits, A. G. J. M. Oude Lansink, and W. van der Werf. 2010. Economic impact assessment in pest risk analysis. Crop. Prot. 29:517–524.

[CIT0035] Soliman, T., M. C. M. Mourits, A. G. J. M. Oude Lansink, and W. van der Werf. 2014. Quantitative economic impact assessment of invasive plant pests: what does it require and when is it worth the effort? Crop. Prot. 69:9–17.

[CIT0036] Sugahara, M., and F. Sakamoto. 2009. Heat and carbon dioxide generated by honeybees jointly act to kill hornets. Naturwissenschaften. 96: 1133–1136. 19551367 10.1007/s00114-009-0575-0

[CIT0037] Takahashi, J., S. Akimoto, S. J. Martin, M. Tumukae, and E. Hasegawa. 2004. Mating structure and male production in the giant hornet *Vespa mandarinia* (Hymenoptera : Vespidae). Appl. Entomol Zool 39:343–349.

[CIT0038] Ugajin, A., T. Kiya, T. Kunieda, M. Ono, T. Yoshida, and T. Kubo. 2012. Detection of neural activity in the brains of Japanese honeybee workers during the formation of a ‘hot defensive bee ball.’ PLoS ONE. 7:1–12. 10.1371/journal.pone.0032902 PMC3303784 22431987

[CIT0039] U.S. Census Bureau. 2019. QuickFacts. Washington, Oregon, Montana, Idaho. Available from https://www.census.gov/quickfacts. Accessed Oct. 2020.

[CIT0040] USDA. 2017. Forest inventory and analysis, fiscal year 2016 business report. U. S. Department of Agriculture, Forest Service, Washington, DC. 84 pp.

[CIT0041] USDA Agricultural Research Service. 2020. Plant hardiness zone map. U. S. Department of Agriculture. Available from https://planthardiness.ars.usda.gov/PHZMWeb/Default.aspx#. Accessed Oct. 2020.

[CIT0042] USDA NASS. 2021. Quick Stats. U. S. Department of Agriculture, National Agricultural Statistics Service. Available from https://data.nal.usda.gov/dataset/nass-quick-stats. Accessed Apr. 2021.

[CIT0043] Vitousek, P. M., H. A. Mooney, J. Lubchenco, and J. M. Melillo. 1997. Human domination of Earth’s ecosystems. Science. 277:494–499.

[CIT0044] Wilson, T. M., J. Takahashi, S. E. Spichiger, I. Kim, and P. van Westendorp. 2020. First reports of *Vespa mandarinia* (Hymenoptera: Vespidae) in North America represent two separate maternal lineages in Washington State, United States, and British Columbia, Canada. Ann. Entomol. Soc. Am. 113:468–472.

[CIT0045] Yoshimoto, J., and T. Nishidia. 2009. Factors affecting behavioral interactions among sap-attracted insects. Ann. Entomol. Soc. Am. 102:201–209.

[CIT0046] Zhu, G., J. Gutierrez Illan, C. Looney, and D. W. Crowder. 2020. Assessing the ecological niche and invasion potential of the Asian giant hornet. Proc. Natl. Acad. Sci. U. S. A. 117: 24646–24648. 32963093 10.1073/pnas.2011441117 PMC7547231

